# Alcohol consumption among adults in Germany: risky drinking levels

**DOI:** 10.17886/RKI-GBE-2017-044

**Published:** 2017-06-14

**Authors:** Cornelia Lange, Kristin Manz, Benjamin Kuntz

**Affiliations:** Robert Koch Institute, Department for Epidemiology and Health Monitoring, Berlin, Germany

**Keywords:** ALCOHOL, RISKY CONSUMPTION, ADULTS, HEALTH MONITORING, GERMANY

## Abstract

Consuming harmful amounts of alcohol is considered a contributing factor in over 200 diseases. Women who drink over 10 g and men who drink more than 20 g of pure alcohol daily are already consuming risky amounts. According to GEDA 2014/2015-EHIS data, 13.8% of women and 18.2% of men consume risky amounts of alcohol at least weekly. The consumption of potentially dangerous levels of alcohol is most widespread in the 45-64 age group. Across all age groups, the prevalence of risky alcohol consumption patterns is higher among highly educated women compared to women with a lower level of education. For men, this pattern only appears in those aged 65 and over. Preventive measures including social and environmental interventions and responsible drinking campaigns should contribute to further reducing risky alcohol consumption among the population.

## Introduction

Alcohol is a potentially addictive psychoactive substance. Consuming harmful levels of alcohol is considered a contributing factor to over 200 diseases; globally, it is among the five key risk factors for disease, impairment and death [1]. In addition, to the impacts harmful amounts of alcohol can potentially have on a person’s health, the World Health Organization (WHO) also highlights the socioeconomic costs for individuals who drink and the consequences for others and society in general [1]. For society, the consequences of people consuming harmful levels of alcohol include the direct costs to the health system, as well as the costs related to the loss of productivity and immaterial costs such as the loss of quality of life. In Germany, alcohol consumption is estimated to cost the economy around EUR 40 billion annually, with around one quarter of this sum being spent directly on the health care system [2, 3]. To reduce harmful levels of consumption among the population, the WHO has developed global and European strategies [4, 5]. In its Global Action Plan for the Prevention and Control of Non-communicable Diseases, the WHO aims to reduce risky consumption by 10 percent by 2025 (with 2010 as a baseline) [6]. Germany’s national health target ‘Reduce alcohol consumption’, initially published in 2015, is in part based on the WHO approach [7]. Statistics for alcohol consumption and trend analyses show alcohol consumption is on the decline in Germany [8]. Nonetheless, this fundamentally positive development cannot conceal the fact that per capita alcohol consumption in Germany remains far higher than the average of EU member states [9] and that Germany’s efforts to implement regulatory measures to reduce harmful levels of drinking are far more tentative than the EU average [10].

**GEDA 2014/2015-EHIS**

**Data holder:** Robert Koch Institute

**Aims:** To provide reliable informa tion about the population’s health status, health-related behaviour and health care in Germany, with the possibility of a European comparison

**Method:** Questionnaires completed on paper or online

**Population:** People aged 18 years and above with permanent residency in Germany

**Sampling:** Registry office sample; randomly selected individuals from 301 communities in Germany were invited to participate

**Participants:** 24,016 people (13,144 women; 10,872 men)

**Response rate:** 26.9%

**Study period:** November 2014 - July 2015

**Data protection:** This study was undertaken in strict accordance with the data protection regulations set out in the German Federal Data Protection Act and was approved by the German Federal Commissioner for Data Protection and Freedom of Information. Participation in the study was voluntary. The participants were fully informed about the study’s aims and content, and about data protection. All participants provided written informed consent.

More information in German is available at www.geda-studie.de

## Indicator

The consumption of risky amounts of alcohol (risky consumption) is a consumption pattern that implies an increased risk for physical and mental health. 10-12 g of pure alcohol daily for women and 20-24 g for men [11, 12] is considered the limit beyond which alcohol poses a health risk. To survey the frequency and amounts of alcohol being consumed [13] the German Health Update 2014/2015-EHIS (GEDA 2014/2015-EHIS) survey applied an instrument from the European Health Interview Survey (EHIS). Based on the Alcohol Use Disorder Identification Test – Consumption Questions (AUDIT-C) [14], the EHIS instrument first ascertains the frequency of alcohol consumption during the past twelve months and then differentiates this information according to the number of standard drinks consumed on weekdays (Monday to Thursday) and weekends (Friday to Sunday). This information makes it possible to define the amount of pure alcohol consumed per day among those who drink every week, as well as the share of people who drink more (or less) than the defined threshold values (consumption of over 10 g of pure alcohol daily for women and over 20 g for men) (the categories are: risky consumption and non-risky consumption). Moreover, the questionnaire registers the number of people who never drink alcohol or who did not drink alcohol during the past 12 months (category non-drinkers), as well as those, who do not drink alcohol every week (category non-weekly consumption). The results are stratified according to gender, age and education and, for risky consumption, according to gender and federal state. A statistically significant difference between groups is assumed where confidence intervals do not overlap.

The analyses are based on the data received from 23,561 participants aged 18 and above (12,913 women and 10,648 men) who provided valid responses on alcohol consumption. Calculations are weighted to account for disparities between the sample and the overall population structure (as of 31 December 2014) with regard to gender, age, type of municipality and education. The type of municipality category reflects the degree of urbanisation and corresponds to the regional distribution in Germany. To ensure comparability of answers, the International Standard Classification of Education (ISCED) was used [15]. A detailed description of the methodology employed in GEDA 2014/15-EHIS is included in German Health Update – New data for Germany and Europe in issue 1/2017 of the Journal of Health Monitoring.

## Results and discussion

**Figure 1:**
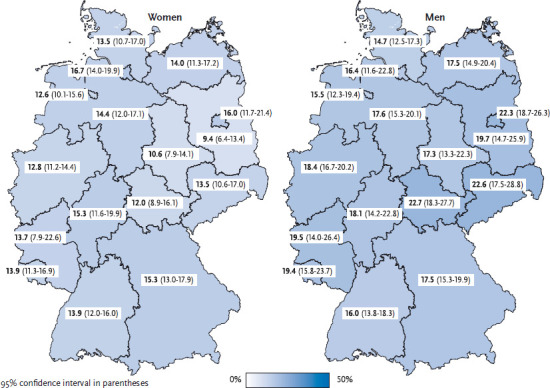
Risky alcohol consumption according to gender and federal state (n=12,913 women; n=10,648 men) Source: GEDA 2014/2015-EHIS

16.9% of women and 10.3% of men never drink alcohol. 13.8% of women and 18.2% of men drink risky amounts of alcohol at least weekly. Consuming risky amounts of alcohol is therefore significantly more widespread among men than women. When data are stratified according to age groups, the prevalence is highest among the 45- to 64-year-olds (17.2% of women and 21.7% of men, Table 1 and Table 2). With the exception of the 30-44 age group, the prevalence of risky consumption is higher among highly educated women than among those with lower levels of education. Results from the German Health Interview and Examination Survey for Adults (DEGS1) [8] and further international surveys [16, 17] provide similar findings. For highly educated men over 65, the same pattern of higher rates of risky consumption is shown as among women. In the 18-64 age group, no notable association between education and risky consumption exists. The prevalence of risky levels of consumption in each of the federal states shows no notable differences to the average prevalence across Germany. Among men, the share of those who consume risky amounts of alcohol is lowest in Schleswig-Holstein (14.7%). With over 22%, this share is significantly higher in Berlin, Saxony and Thuringia. The prevalence of risky levels of consumption among women is significantly higher in Hamburg (16.7%) than in Brandenburg (9.4%) (Figure 1).

Instruments such as the index used in GEDA 2014/2015-EHIS (based on the EHIS instrument) to measure frequency and amounts of alcohol consumed rely on the self-assessment of interviewees, whereby memory, the correct judgement of glass sizes, as well as social pressure to give certain answers (known as social desirability bias) may all influence results. Consuming larger amounts of alcohol on one occasion, otherwise referred to as heavy episodic drinking, is another risky form of alcohol consumption; however, this behaviour is not considered in this article. Specific analysis of heavy episodic drinking can be found on the Fact sheet Alcohol consumption among adults in Germany: heavy episodic drinking. As the survey tool and indicator are no longer the same as in previous GEDA survey waves, no conclusions can be drawn concerning trends. Results from other surveys, however, can help contextualise the results. According to data from the Epidemiological Survey of Substance Abuse 2015 for the 18-64 age group, 13.4% of women and 17.0% of men reported drinking risky amounts of alcohol during the past 30 days (consumption of over 12 g of pure alcohol daily for women and over 24 g for men) [18]. According to DEGS1 results, 13.1% of women and 18.5% of men in the 18-79 age group reported drinking over 10 g (women) and 20 g (men) of pure alcohol per day on average during the past four weeks [8]. The association between social status and alcohol consumption, which the survey analysed, is comparable to the results suggested by GEDA data. Even though the corresponding survey indicators are not entirely congruent, the results are of the same magnitude: around one in seven women and nearly one in five men consume amounts of alcohol that increase the risk of negative impacts on health. Public policies with the purpose of reducing alcohol consumption are therefore as important as publicly advocating responsible drinking. Through its campaign ‘Kenn dein Limit – Bewusst genießen, im Limit bleiben’ (Respect your limits – enjoy responsibly and stay below the limit), the Federal Centre for Health Education offers information on low-risk forms of drinking (https://www.kenn-dein-limit.de/).

## Key statements

- 14% of women and 18% of men drink harmfully high levels of alcohol.
- The prevalence of risky alcohol consumption is highest for women and men in the 45-64 age group (17% of women, 22% of men).
- Rates of risky alcohol consumption are higher among highly educated women than among those with a lower level of education, with the same applying to men aged over 65.

## Figures and Tables

**Table 1:** Alcohol consumption among women according to age and educational status (n=12,913) Source: GEDA 2014/2015-EHIS

Women	Non-drinkers		Non-weekly consumption		Non-risky consumption		Risky consumption	
	%	(95% CI)	%	(95% CI)	%	(95% CI)	%	(95% CI)
**Women total**	**16.9**	**(15.9-17.8)**	**47.1**	**(45.9-48.4)**	**22.2**	**(21.2-23.1)**	**13.8**	**(13.0-14.7)**
**18-29 Years**	15.0	(12.8-17.4)	56.9	(54.1-59.6)	15.3	(13.6-17.3)	12.8	(11.1-14.7)
Low education	26.5	(20.4-33.6)	55.5	(48.6-62.2)	8.9	(5.9-13.4)	9.1	(6.1-13.4)
Medium education	11.7	(9.6-14.2)	59.7	(56.6-62.7)	15.5	(13.3-18.0)	13.1	(11.0-15.5)
High education	10.7	(7.9-14.2)	47.5	(42.4-52.7)	24.4	(20.1-29.2)	17.4	(14.0-21.4)
**30-44 Years**	16.5	(14.8-18.3)	52.0	(49.8-54.3)	20.5	(18.6-22.4)	11.0	(9.5-12.8)
Low education	27.8	(21.6-35.0)	53.1	(45.9-60.2)	11.4	(7.7-16.6)	7.7	(4.5-12.7)
Medium education	15.0	(12.9-17.4)	54.1	(51.1-56.9)	20.3	(17.9-22.9)	10.6	(8.9-12.7)
High education	13.6	(11.6-15.9)	46.7	(43.5-50.0)	25.8	(22.9-28.8)	13.9	(11.2-17.2)
**45-64 Years**	12.5	(11.5-13.7)	44.2	(42.5-45.9)	26.1	(24.7-27.6)	17.2	(15.9-18.6)
Low education	21.5	(18.0-25.3)	45.0	(40.4-49.6)	22.1	(18.1-26.5)	11.5	(8.9-14.7)
Medium education	11.5	(10.2-13.0)	46.6	(44.5-48.7)	25.5	(23.7-27.4)	16.3	(14.7-18.1)
High education	7.9	(6.5-9.6)	35.4	(32.7-38.1)	31.5	(28.8-34.2)	25.3	(22.8-28.0)
**≥ 65 Years**	24.3	(22.3-26.4)	40.9	(38.8-43.1)	22.5	(20.6-24.5)	12.2	(10.7-13.9)
Low education	32.1	(28.7-35.6)	41.2	(37.6-44.8)	18.0	(15.2-21.2)	8.8	(6.9-11.1)
Medium education	19.5	(17.0-22.2)	41.6	(38.3-45.1)	25.6	(22.8-28.5)	13.3	(11.2-15.9)
High education	17.4	(12.9-23.1)	35.9	(31.1-41.1)	25.3	(21.2-30.0)	21.3	(17.7-25.5)
**Total (women and men)**	**13.7**	**(13.0-14.4)**	**38.9**	**(38.0-39.8)**	**31.4**	**(30.6-32.3)**	**16.0**	**(15.3-16.6)**

CI=confidence interval

**Table 2:** Alcohol consumption among men according to age and educational status (n=10,648) Source: GEDA 2014/2015-EHIS

Men	Non-drinkers		Non-weekly consumption		Non-risky consumption		Risky consumption	
	%	(95% CI)	%	(95% CI)	%	(95% CI)	%	(95% CI)
**Men total**	**10.3**	**(9.6-11.2)**	**30.3**	**(29.2-31.5)**	**41.1**	**(39.8-42.5)**	**18.2**	**(17.3-19.1)**
**18-29 Years**	10.5	(8.5-12.8)	39.2	(36.1-42.4)	33.1	(30.1-36.2)	17.3	(15.2-19.6)
Low education	14.1	(9.9-19.7)	43.2	(36.4-50.3)	24.3	(18.7-30.9)	18.4	(13.4-24.9)
Medium education	10.1	(7.9-12.7)	39.0	(35.4-42.6)	33.4	(29.8-37.2)	17.6	(15.1-20.4)
High education	5.4	(3.0-9.4)	33.5	(28.2-39.2)	47.3	(41.3-53.4)	13.8	(10.2-18.4)
**30-44 Years**	9.4	(7.9-11.1)	36.9	(34.3-39.5)	40.3	(37.7-42.8)	13.5	(11.8-15.4)
Low education	22.8	(16.5-30.8)	33.5	(26.1-41.8)	26.8	(20.1-34.6)	16.9	(11.6-23.9)
Medium education	8.6	(6.7-10.9)	40.1	(36.4-44.0)	38.5	(34.9-42.2)	12.8	(10.5-15.5)
High education	5.4	(3.9-7.4)	32.1	(28.6-35.8)	49.6	(45.8-53.4)	13.0	(10.6-15.8)
**45-64 Years**	10.0	(9.0-11.1)	25.3	(23.7-27.0)	43.0	(41.1-44.9)	21.7	(20.2-23.3)
Low education	16.5	(12.8-21.0)	31.2	(26.6-36.1)	30.9	(25.9-36.4)	21.4	(17.7-25.7)
Medium education	11.0	(9.6-12.6)	26.5	(24.2-28.9)	41.7	(39.0-44.4)	20.9	(18.6-23.3)
High education	6.1	(4.9-7.4)	21.1	(19.0-23.4)	49.4	(46.6-52.3)	23.4	(21.3-25.5)
**≥ 65 Years**	11.8	(10.4-13.4)	24.6	(22.6-26.8)	45.6	(43.3-47.8)	17.9	(16.4-19.6)
Low education	17.3	(13.9-21.3)	30.1	(25.3-35.3)	39.6	(34.2-45.3)	13.0	(9.4-17.7)
Medium education	13.3	(11.2-15.9)	24.6	(21.7-27.8)	45.2	(41.9-48.5)	16.8	(14.5-19.4)
High education	7.0	(5.6-8.7)	21.9	(19.4-24.7)	49.0	(45.9-52.1)	22.1	(19.6-24.8)
**Total (women and men)**	**13.7**	**(13.0-14.4)**	**38.9**	**(38.0-39.8)**	**31.4**	**(30.6-32.3)**	**16.0**	**(15.3-16.6)**

CI=confidence interval
